# Low Dose Ketamine Preconditions Astrocyte Mitochondria to Achieve Antidepressant Efficacy via Adenosine, Humanin, and Melatonin Upregulation and Efflux

**DOI:** 10.31083/AP54760

**Published:** 2026-08-24

**Authors:** George Anderson

**Affiliations:** ^1^CRC Scotland & London, SW1V 1PG London, UK

**Keywords:** Ketamine, depression, melatonin, adenosine, humanin, treatment, suicide, STAT3, NF-κB, mitochondria

## Abstract

Understanding the physiological changes induced by ketamine in mood disorders, while reducing its side effects, is likely to contribute to a more comprehensive understanding of the physiology underlying mood disorders, as well as to the development of faster-acting and more efficacious antidepressant treatments. An extensive review of the literature on ketamine and the pathophysiology of depression indicates that ketamine's antidepressant efficacy has classically been attributed to noncompetitive antagonism of the n-methyl-d-aspartate receptor (NMDAR) on the neuronal postsynaptic membrane, thereby reducing excessive Ca²⁺ influx through the NMDAR channel. However, recent evidence suggests that ketamine's antidepressant efficacy is mediated not by synaptic NMDAR antagonism but rather by its direct effects on mitochondrial function, arising from its amphiphilic structure. It is proposed that astrocyte mitochondria represent the primary target of ketamine, with the broader effects induced by ketamine occurring downstream of the optimization of astrocyte mitochondrial function and, consequently, astrocyte function. Developmental stress and trauma are proposed to differentially prime specific regions of the central nervous system, rendering them more susceptible to subsequent stressors through the epigenetic regulation of astrocytes. This process increases astrocyte reactivity and dysregulates astrocyte mitochondrial function in response to subsequent stressors, while also increasing blood-brain barrier permeability within these regions. At stress-vulnerable sites, ketamine may upregulate adenosine, humanin, and melatonin, thereby restoring astrocyte function and attenuating inflammatory activity within the local astrocytic microenvironment, including microglia, neurons, and oligodendrocytes. Ketamine's modulation of mitochondrial function is proposed to be mediated via NMDARs located on the inner mitochondrial membrane, leading to alterations in mitochondrial ionic regulation that enhance astrocyte mitochondrial resilience to stress, possibly through a preconditioning mechanism. Ketamine-induced increases in melatonin and humanin are proposed to suppress microglial activation, promote white matter remyelination, and restore neuronal activity, as well as patterned intercellular and interregional communication. This hypothesis-driven overview evaluates ketamine's capacity to restore astrocyte mitochondrial function and thereby counteract the consequences of developmental stress and trauma that underlie vulnerability to subsequent stress-induced depression.

## Main Points

1. Ketamine efficacy highlights the role of astrocyte mitochondria as core processes in depression pathoetiology and pathophysiology.

2. Developmental/childhood stress/trauma, via increased cortisol at the glucocorticoid receptor, epigenetically regulates astrocyte mitochondria to increase depression susceptibility to subsequent stress/trauma.

3. Ketamine acts on astrocyte mitochondria by inhibiting the n-methyl-d-aspartate receptor (NMDAR) on the astrocyte inner mitochondrial membrane and by acting as a positive allosteric modulator of the brain-derived neurotrophic factor (BDNF) receptor, tropomyosin receptor kinase (Trk)B.

4. Ketamine is proposed to precondition astrocyte mitochondria by altering ionic regulation to negate the consequences of developmental stress/trauma.

5. Ketamine is proposed to increase astrocyte melatonin and humanin to re-establish the blood-brain barrier (BBB), the glymphatic system and neuronal function by acting on the mitochondria of microglia, neurons, oligodendrocytes and endothelial cells.

## 1. Introduction

As an anesthetic, fast-acting antidepressant, psychotomimetic and drug of abuse, ketamine and its active metabolites, norketamine and hydroxynorketamine (HNK), can have diverse effects classically attributed to ketamine’s capacity as a non-competitive n-methyl-d-aspartate receptor (NMDAR) antagonist at the neuronal post-synaptic membrane [1,2]. Ketamine is amphiphilic and therefore has diverse systemic effects, including decreasing gut dysbiosis/permeability whilst increasing the gut microbiome-derived short-chain fatty acid, butyrate [3], which contribute to its suppression of systemic and central nervous system (CNS) inflammation [4]. Ketamine also directly regulates immune cells [5,6]. Ketamine’s diverse effects suggest modulation of core cellular (mitochondrial) and systemic (circadian/inflammation/oxidative stress) processes [7], indicative of a complexity beyond simple post-synaptic NMDAR antagonism. Ketamine’s rapid antidepressant effects may therefore be complex and possibly variable given the wide pathophysiological heterogeneity in major depressive disorder (MDD) [8] and/or ketamine may be acting on a common ‘hub’ upon which diverse MDD pathophysiological processes converge.

Although classically conceptualized as arising from monoamine dysregulation [9], a complexity of pathoetiologcal and pathophysiological processes contribute to MDD in a given individual [8], including prenatal/postnatal stress, early epigenetic alterations in stress responsivity, abusive and traumatic events as well as alterations in the gut microbiome/permeability, immune dysregulation, circadian disruption, oxidative stress, autonomic nervous system and mitochondrial function [10,11,12,13,14,15]. Current treatment still typically focuses on monoamine modulation, although novel treatments are emerging, including repetitive transcranial magnetic stimulation (rTMS), transcranial direct current stimulation (tDCS), and deep-brain stimulation (DBS) [16]. However, there are still unacceptably high levels of suicide and suicide attempts [17]. The capacity of ketamine/esketamine/HNK to rapidly shift many people from a depressed, suicidal state is therefore of considerable clinical importance and likely to enlighten the physiological understanding of mood regulation, especially if ketamine is acting on a core ‘hub’.

Intravenous ketamine (and intranasal esketamine) antidepressant effects are classically proposed to be primarily mediated via synaptic NMDAR-NR2D subunit inhibition, whilst its psychotomimetic effects are proposed to be mediated via neuronal NMDAR NR2A/2B subunits inhibition [18]. This provides a simple model of MDD and its treatment as arising from alterations in glutamatergic neuronal function, as determined by NMDAR subunit composition and activation [19]. This is challenged by recent preclinical data showing ketamine’s, and other fast-acting antidepressants’, efficacy to be mediated via direct effects on mitochondrial metabolism, coupled with increased adenosine directly acting on mitochondria, especially in the medial prefrontal cortex (mPFC) [20].

Classical neuroncentric conceptualizations of brain function are challenged by data highlighting the powerful role of astrocytes and microglia, as well as circadian and systemic processes, in shaping neuronal function [21,22]. Sleep can be conceptualized as a period of mitochondrial restoration whereby daytime stress driven mitochondrial fission is shifted to melatonin/sirtuin-3 driven mitochondrial fusion at night [23]. Circadian processes are therefore intimately intertwined with mitochondria regulation and how mitochondria are prepared for the coming day.

This article places ketamine effects within a framework that highlights the importance of mitochondrial function in MDD pathophysiology, with developmental stress/trauma increasing MDD susceptibility to subsequent stress via the epigenetic consequences of cortisol’s effects at the glucocorticoid receptor (GR)-α. It is proposed that low dose ketamine has initial toxic/challenging effects in mitochondria that ‘preconditions’ mitochondria to enhance mitochondrial function and stress resilience. As ketamine is amphiphilic and crosses the blood-brain barrier (BBB), the first brain cell encountered and entered is highly likely to be the astrocytic end-feet that enwrap capillary endothelial cells. Within astrocytes, ketamine can inhibit the astrocyte plasma membrane NMDAR. Ketamine can also readily diffuse over the mitochondrial bilipid membrane and/or can be actively transported by the organic cation transporter (OCT)3 that is present on astrocyte mitochondrial membranes [24]. This article therefore re-frames ketamine’s antidepressant effects, proposing that these arise from the regulation of astrocyte mitochondria, especially at sites primed by developmental stress/trauma, including the mPFC, where astrocyte dysregulation is a core aspect of MDD pathophysiology. The capacity of ketamine to increase local mitochondrial melatonin, including by increasing cyclic adenosine monophosphate (cAMP)/protein kinase A (PKA) [25], that is present in all mitochondria containing cells [26] allows ketamine to have anti-inflammatory, antioxidant and mitochondria optimizing effects at least partly via astrocyte autocrine and paracrine melatonin.

This hypothesis-driven overview evaluates whether ketamine resets astrocyte mitochondrial function. It is proposed that ketamine ‘resets’ the developmental effects of stress/trauma by acting on astrocyte mitochondria at particular CNS sites, with the diverse bodies of data on ketamine’s modulation of neuronal and microglia function downstream of its effects on astrocyte mitochondria.

First, data are reviewed on the role of glia, especially astrocytes in the mPFC, in MDD.

## 2. Ketamine, Astrocytes, Microglia and Mitochondria

As noted, the proposed mode of ketamine’s antidepressant efficacy have been many and various [1,2,3,4,5,6,7], with alterations in neuronal plasma membrane NMDAR Ca^2+^ influx proposed to underpin many of the diverse changes that ketamine can induce in the CNS, including via compensatory α-amino-3-hydroxy-5-methyl-4-isoxazolepropionic acid receptor (AMPAr) upregulation and AMPAr derived smaller Ca^2+^ influx [27,28]. As astrocytes have a powerful role in controlling synapse development and maintenance, including AMPAr insertion in synapses [29,30], ketamine’s upregulation of synaptic AMPAr may be mediated via astrocytes. However, as well as astrocytes [31], ketamine can also have direct effects on other CNS cells, including microglia, oligodendrocytes and neurons directly as well as indirect effects via initial effects in astrocytes, providing a complexity of dynamic interactions among CNS cells in the course of ketamine’s antidepressant efficacy [32]. However, ketamine’s initial CNS effects in astrocytes are proposed to provide a co-ordinated regulation of changes in other CNS cells upon which ketamine may also have direct effects.

Astrocytes modulate diverse CNS conditions [21,33], including MDD [34]. Astrocytes are multi-tasking cells that regulate cerebral vasodilation, BBB permeability, the glymphatic system, neurotransmitter uptake and recycling, K^+^ uptake and recycling, enwrap most synaptic clefts to modulate neurotransmission, release adenosine triphosphate (ATP) and glutamate to activate neurons, as well as providing neurons with lactate for energy provision, which neurons readily convert into pyruvate [35]. Astrocytes are also ‘immune-type’ cells capable of antigen presentation, with a gradation of reactive and quiescent phenotypes driven by a shift from an nuclear factor kappa-light-chain-enhancer of activated B cells (NF-κB) p65/p50 dimer to an NF-κB c-Rel/p50 dimer, respectively [36]. Given such diverse astrocytic functions, neuronal activity can be conceptualized as a form of ‘immune-to-immune’ communication, including ‘glia-to-glia’ communication within the CNS [37]. Consequently, ketamine’s modulation of astrocytes can impact on many key CNS processes pertinent to MDD and ketamine’s rapid antidepressant efficacy [34]. As astrocytic end-feet, along with pericytes, enwrap the blood vessels to form the BBB, the first cell encountered by ketamine in the CNS is highly likely to be an astrocyte.

Astrocytes express a plasma membrane NMDAR, the knockout of which alters astrocyte and neighboring neuron function in preclinical models [38]. The detrimental effects of astrocyte plasma membrane NMDAR knockout are prevented by ATP supplementation, suggesting purinergic and/or metabolic effects are regulated by the astrocyte NMDAR [38]. ATP may be converted sequentially by cluster of differentiation (CD)39 and CD73 to form adenosine, which affords protection to challenged mitochondria, suggesting that the astrocyte NMDAR is intimately linked to mitochondrial ATP/adenosine regulation [20]. Astrocyte NMDAR activation is strongly associated with presynaptic glutamate release, thereby preparing astrocytes to upregulate the energy production (ATP, oxidative phosphorylation (OXPHOS) and glycolysis) that is required in the course of neuronal activity, including increased lactate provision to neurons. The astrocyte plasma membrane NMDAR also regulates mitochondrial positioning and mitophagy [39,40,41], thereby optimizing mitochondrial function and positioning to deal with local sites of high energy demand, such as glutamatergic overspill at synapses [41]. The initial effects of circulating ketamine in the CNS may therefore include alterations in the regulation of astrocyte plasma membrane NMDAR associated processes, including astrocyte metabolism. This shifts the site of ketamine effects from the neuronal to astrocyte plasma membrane but with significant consequences in both cell types.

Ketamine regulates many aspects of astrocyte function, including: elevating intracellular cAMP, attenuating stimulus induced Ca^2+^, regulating exocytotic gliotransmitter release, stabilizing a narrow vesicle fusion pore to attenuate cargo discharge or vesicle recycling, suppressing the cytoplasmic mobility of vesicles to decrease inward rectifying potassium channel (Kir4.1) delivery to the plasma membrane, modulating astrocyte K^+^ buffering, and rapidly redistributing astrocyte (but not neuronal) plasma membrane cholesterol, thereby altering lipid rafts and plasma membrane organizational structure, reviewed in [31]. Ketamine also alters astrocyte morphology and reverses many of the changes seen in MDD, including astrocyte cell body size as well as the length and number of astrocytic processes, whilst also altering norepinephrine interactions with astrocytes, which are proposed to decrease futility and increase perseverance to stress in preclinical MDD models [42]. Ketamine can therefore directly regulate many aspects of astrocyte function, which may be determined by non-competitive antagonism of the astrocyte plasma membrane NMDAR and/or mitochondrial inner membrane NMDAR to modulate mitochondrial function. Ketamine’s diverse changes in astrocyte structure and function require careful temporal monitoring over the short time of ketamine’s actions as the rapid changes in plasma membrane structure and lipid raft organization may be indicative of a preconditioning-like stressor effect.

Ketamine also inhibits the pyroptosis-associated NACHT, LRR, and PYD domains-containing protein 3 (NLRP3) inflammasome in astrocytes, thereby suppressing NLRP3-induced IL-1β and IL-18 as well as glymphatic system dysfunction [43]. Whether NLRP3 inflammasome suppression involves the attenuation of phosphorylated Signal transducer and activator of transcription 3 (STAT3)^Ser727^ mitochondrial translocation, which can induce NLRP3 to translocate adjacent to mitochondria in other cell types [44], requires future investigation in astrocytes. As noted [1,2,3,4,5,6,7], many processes in both astrocytes and neurons are proposed to underpin ketamine’s rapid antidepressant effects, including a sustained antidepressant effect of ketamine being mediated by the astrocyte transfer of sigma-1 receptors to neurons, via a process involving activation of the mitochondrial protein, Translocator Assembly and Maintenance protein 41(TAM41) mitochondrial translocator assembly and maintenance homolog (TAMM41) via a TAMM41-cardiolipin-exosomes axis [45], which the same research lab proposed to underpin the benefits of fluvoxamine in preclinical models of epilepsy [46]. Overall, the correlated effects of ketamine on processes classically linked to MDD and suboptimal brain function are many and various, especially in the regulation of the wide-ranging processes regulated by astrocytes.

Ketamine, over a variety of different models, including anaesthesia, sepsis and depression, elevates the brain-derived neurotrophic factor (BDNF) receptor, tropomyosin kinase receptor (Trk)B, sometimes in the absence of any increase in BDNF levels [47]. By inhibiting NMDAR activation ketamine can indirectly increase BDNF to activate TrkB-full length (TrkB-FL). This is another mechanism proposed to mediate ketamine’s antidepressant effects, including by BDNF/TrkB increasing synaptic plasticity and neurogenesis [48,49] as well as by inducing intracellular signaling pathways that increase mitochondrial function and ATP production [50]. BDNF activation of TrkB-FL can also lead to their translocation to mitochondria, which regulates mitochondria transport and distribution [50]. BDNF/TrkB can be increased in many cell types, including astrocytes and neurons, with TrkB-FL activation typically having trophic and/or proliferative effects as well as regulating the astrocyte excitatory amino acid transporter (EAAT)2 levels [51]. However, in the hypothalamic paraventricular nucleus (PVN) BDNF/TrkB-FL are major drivers of stress-induced synaptic plasticity, which includes increasing corticotropin-releasing hormone (CRH) that initiates and upregulates the hypothalamic-pituitary-adrenal (HPA) axis to enhance cortisol production [52,53]. Sites of BDNF/TrkB-FL induction and activation in the CNS can therefore have distinct effects, which may contribute to the diverse effects of ketamine.

In anaesthesia models, ketamine increases TrkB but not BDNF, via glycogen synthase kinase (GSK)-3β regulation [47]. Interestingly, ketamine binds to TrkB and may directly activate and/or positively allosterically modulate TrkB to enhance BDNF effects, including by disrupting TrkB-FL interactions with the adaptor protein complex 2 (AP-2), which is crucial for TrkB-FL to be internalized [54]. Notably, TrkB can be expressed on the plasma membrane and mitochondrial membrane and may be in full-length form or truncated, the most common truncated form being TrkB-T1, which is highly expressed in astrocytes [55]. TrkB can also be activated by the immediate precursor to melatonin in the tryptophan-melatonin pathway, namely NAS [56]. NAS also increases BDNF, as shown in the rodent hippocampus [57]. As ketamine increases cAMP/protein kinase A (PKA) and local melatonin production [25], it will also increase NAS, not all of which is converted to melatonin, with NAS activating TrkB as well as inducing BDNF. This requires investigation in astrocytes, where the melatonergic pathway has been shown to be present [58], suggesting that variations in the NAS/melatonin ratio, as in the pineal gland and other medical conditions [59], may be a relevant aspect of ketamine’s astrocyte mitochondria regulation via ketamine upregulation of NAS that activates TrkB, the activation of which is further enhanced by ketamine’s positive allosteric modulation of TrkB.

**Fig. 1.**
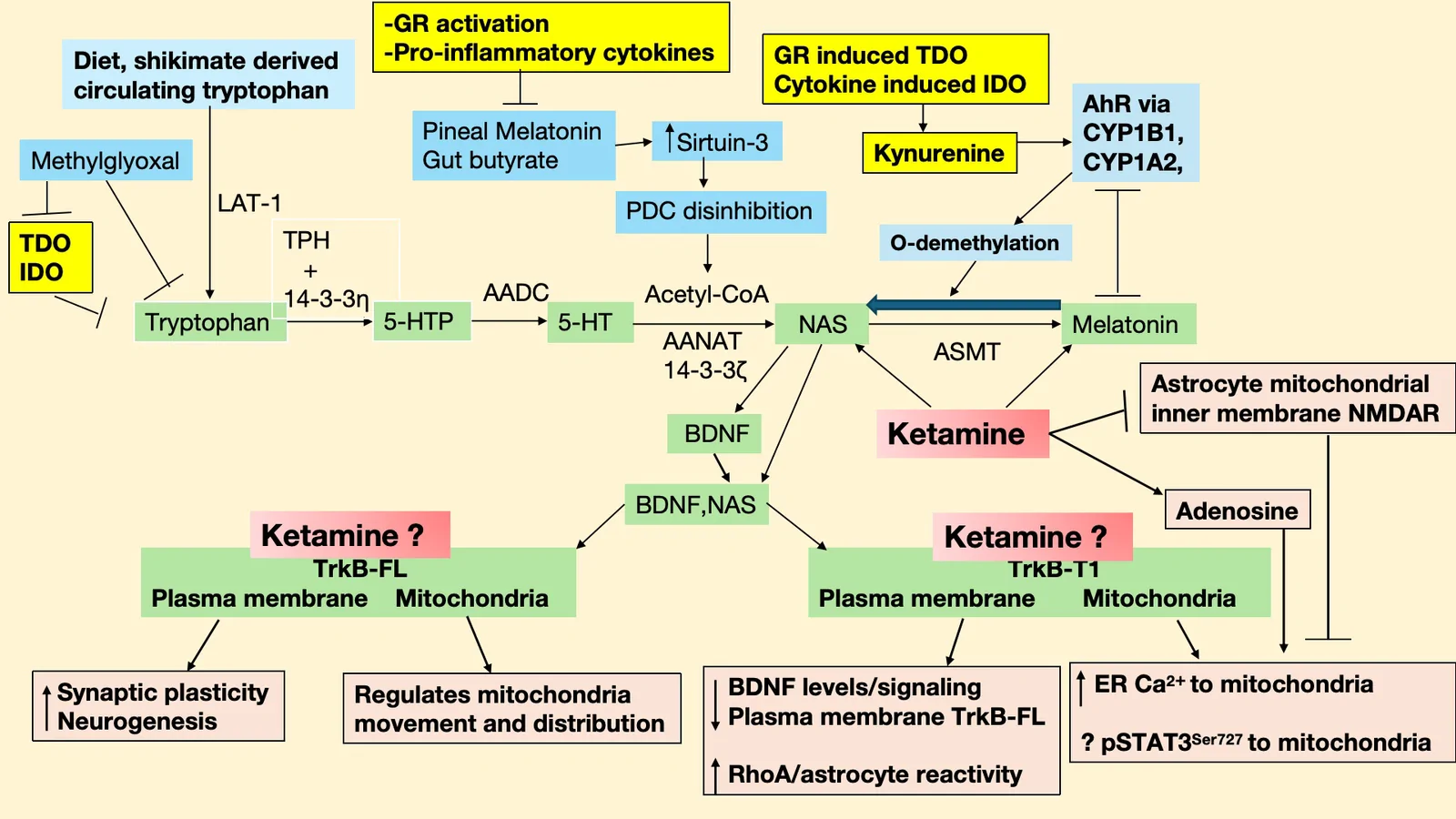
**Shows ketamine to be intimately linked to BDNF and TrkB effects**. Ketamine can increase not only mitochondrial melatonin but also NAS. Factors regulating the tryptophan-melatonin pathway (blue and yellow shade) will therefore influence ketamine effects and all show alterations in MDD that attenuate tryptophan-melatonin pathway activation. Factors influencing the NAS/melatonin ratio such as AhR activation (blue shade, top right) can O-demethylate melatonin to its immediate precursor, NAS, which increases BDNF, both of which, like ketamine, can bind TrkB-FL and TrkB-T1, whether located on the plasma membrane and/or mitochondrial membrane. The consequences of TrkB activation are therefore variable dependent upon TrkB-FL vs TrkB-T1 isoforms and site of localization. Consequently, ketamine may have very distinct activation/allosteric effects that are dependent on TrkB isoform and localization, with relevance to TrkB in neurons and astrocytes as well as other CNS and systemic cells. Ketamine’s activation of astrocyte TrkB-T1 increases ER Ca^2+^ influx which may be further dysregulated by concurrent ketamine suppression of the astrocyte inner mitochondrial NMDAR and ketamine’s upregulation of adenosine, which increases mitochondria Ca^2+^. The capacity of TrkB-T1 to increase STAT3 may indicate an induction of pSTAT3^Ser727^, which can translocate to MAMs to decrease ER Ca^2+^ efflux to mitochondria. Ca^2+^ regulation at astrocyte MAMs may therefore be a core aspect of mood regulation and fast-acting antidepressant efficacy via a more optimal small increase in Ca^2+^ in astrocyte mitochondria. Abbreviations: 5-HT, serotonin; 5-HTP, 5-hydroxytryptophan; AADC, aromatic-L-amino acid decarboxylase; AANAT, aralkylamine N-acetyltransferase; acetyl-CoA, acetyl-coenzyme A; AhR, aryl hydrocarbon receptor; ASMT, N-acetylserotonin O-methyltransferase; BDNF, brain-derived neurotrophic factor; CNS, central nervous system; CYP, cytochrome P450; ER, endoplasmic reticulum; GR, glucocorticoid receptor; IDO, indoleamine 2,3-dioxygenase; MDD, major depressive disorder; MAMs, mitochondria-associated membranes; NAS, N-acetylserotonin; NMDAR, n-methyl-d-aspartate receptor; PDC, pyruvate dehydrogenase complex; pSTAT3, phosphorylated Signal transducer and activator of transcription 3; RhoA, Ras homolog family member A; TDO, tryptophan 2,3-dioxygenase; TrkB-FL, tropomyosin receptor kinase B-full length; TrkB-T1, tropomyosin receptor kinase B-truncated. ?: indicates that requires experimental validation.

TrkB-T1 and other truncated isoforms, due to the lack of an intracellular tail, have classically been described as dominant-negative inhibitors of TrkB-FL, including by forming a heterodimer with TrkB-FL and by decreasing BDNF and plasma membrane TrkB-FL expression [60]. However, TrkB-T1 can also regulate transcription, which may be especially important in astrocytes where TrkB-T1 is the predominant isoform [61]. These authors showed that the truncated intracellular domain of TrkB-T1 interacts with Rho guanine nucleotide dissociation phosphorylated (Rho GDP) dissociation inhibitor 1 (RhoGDI1), which inhibits the activation of the small guanosine triphosphatase (GTPase), Ras homolog family member A (RhoA). However, when BDNF binds to TrkB-T1, the interaction of TrkB-T1 with RhoGDI1 is broken leading to RhoA signaling, which regulates the cytoskeleton, typically leading to process retraction and therefore an aspect of astrocyte activation [61]. The effects of TrkB-T1 at mitochondria still await full investigation. Data currently indicate that mitochondrial TrkB-T1 upregulates Ca^2+^ from the endoplasmic reticulum (ER) suggesting the presence of TrkB-T1 at mitochondria-associated membranes (MAMs), whilst TrkB-T1 can also act as a dominant-negative regulator of BDNF/TrkB-FL at mitochondria [62]. Whether TrkB-T1 interacts with mitochondria translocating pSTAT3^Ser727^ at MAMs requires clarification given data in glioma showing TrkB-T1 to augment STAT3 signaling [63], with pSTAT3^Ser727 ^, in contrast to TrkB-T1, repressing ER Ca^2+^ signaling to mitochondria. As the mitochondria-optimizing effects of ketamine, like other astrocyte regulators, requires an increase in mitochondria Ca^2+^ influx, the interactions of TrkB-T1 and pSTAT3^Ser727^ at MAMs, as with the sigma-1 receptor activation by hallucinogenic antidepressants at MAMs, may be a core aspect of their antidepressant efficacy. Ketamine effects may therefore modulate the diverse consequences of TrkB activation at both the plasma membrane and mitochondrial membrane, with relevance to core aspects of MDD pathophysiology, as shown in Fig. 1.

Heightened ER Ca^2+^ influx by TrkB-T1 at MAMs can upregulate or dysregulate astrocyte mitochondrial function. As ketamine may not only increase ER Ca^2+^ influx at MAMs by TrkB-T1 but also attenuate the ER Ca^2+^ regulation by the astrocyte inner membrane NMDAR, ketamine may initially and transiently act to initiate classical stress-like, energy requiring signaling in mitochondria via modulating core mitochondrial processes driven by variations in mitochondria Ca^2+^ regulation, as shown in Fig. 1. This is a significant challenge for mitochondria and cellular function as dysregulated mitochondrial Ca^2+^ influx is a major driver of cell death by apoptosis and necrosis [64]. These transient stress-like effects of low-dose ketamine are given support by the effects of higher ketamine doses and prolonged exposure on mitochondrial function, which can lead to astrocyte and microglia reactivity that is neurotoxic to neurons and drives their elimination as well as being neurotoxic in glia [65]. Higher dose ketamine also seems to be associated with an increased extravasation of CD4^+^ T cells into the hippocampus and PFC, implicating changes in the wider systemic immune system in the CNS at higher ketamine dosage [66]. Repeat ketamine administration is also associated with the development of psychotic-like symptoms/dissociation, being proposed to model schizophrenia, including by increasing S100 calcium-binding protein B (S100B) and astrocyte reactivity [67]. Low-dose ketamine effects via astrocyte TrkB-T1 and inner membrane NMDAR may therefore be influencing core aspects of mitochondrial function by initiating a transient stress-like response that requires a survival-linked rapid plasticity response to optimize mitochondrial ionic regulation. This is the essence of preconditioning, whereby a transient non-lethal stressor leads to a ‘bounce back’ strengthening that increases resilience to subsequent stressors. It is proposed here that it is such preconditioning-like changes in astrocyte mitochondria that underpin ketamine’s rapid changes in astrocyte lipid raft rearrangement [31]

## 3. Ketamine, Depression and Mitochondria

Alterations in mitochondrial function are core features of MDD [68,69], with many diverse aspects of mitochondrial function associated with MDD and its treatment. Importantly, ketamine is amphiphilic and can readily cross plasma membranes to gain access to the cellular cytoplasm, which allows ketamine to target its classical site within the intracellular domain of the plasma membrane NMDAR pore, whether in neurons or astrocytes [70]. When crossing the BBB ketamine initially encounters astrocytes, indicating that its first site of modulation within the CNS may be on the astrocyte NMDAR. However, being amphiphilic ketamine may also cross the astrocyte mitochondrial bilipid membrane to gain access to the mitochondrial matrix and/or may be transported into mitochondria by the OCT3, which is expressed on the astrocyte [24] and neuronal mitochondrial membrane, with OCT1 also expressed on the neuronal mitochondrial membrane [71].

As noted, MDD preclinical models show ketamine to directly modulate and reprogram mitochondrial function in isolated neuronal mitochondria, which the authors suggest indicates NMDAR-independent effects of ketamine [20]. These authors propose that ketamine acts selectively in the mPFC and hippocampus to increase adenosine and thereby decrease the ATP/adenosine ratio. This is accompanied by significant shifts in mitochondrial metabolism, including a dose dependent decrease in pyruvate utilization and decreased fumarate, malate and aspartate levels [20]. This study also shows that novel ketamine-derived compounds can show greater adenosine enhancing efficacy than ketamine, suggesting scope for future pharmaceutical development [20]. This investigation strongly indicates that the adenosine enhancing/antidepressant effects of ketamine are independent of the plasma membrane NMDAR [20]. Adenosine’s antidepressant effects are proposed to be via A_1_ receptor activation induced Gi-coupled signaling and the Gs-coupled A_2A_ receptor which is proposed to increase BDNF to promote synaptic plasticity [20]. As Gs-coupled A_2a_ receptor activation increases cAMP/PKA, ketamine’s induction of adenosine would be expected to initiate the melatonergic pathway. Local adenosine infusion in the mPFC or optogenetic activation of mPFC astrocytes affords antidepressant effects via CD73-dependent ATP-to-adenosine conversion [20]. These authors suggest that adenosine mediates the antidepressant effects of ketamine (as with electro-convulsive therapy (ECT) and induced hypoxia) via modulating mitochondrial metabolism, whilst they propose that the neuronal plasma membrane NMDAR effects drive the psychotomimetic effects of ketamine [20].

Mitochondrial dysfunction/stress is long appreciated to upregulate adenosine including under conditions of inflammation, hypoxia and ischemia, with adenosine then acting intercellularly to increase mitochondrial biogenesis and ATP [72,73]. Adenosine affords protection, including by acting on the adenosine A_3A _receptor on the mitochondrial outer membrane to increase ATP production [74]. Ketamine effects, by acting as an initial transient stressor, includes adenosine upregulation that have intracellular and extracellular effects that upregulate mitochondrial metabolism. Astrocytes are a major source of CNS adenosine primarily from ATP release, which is then converted to adenosine by CD39 and ecto-5-nucleotidases, such as CD73. Under conditions of inflammation and hypoxia/ischemia, astrocytes also release adenosine via the equilibrative nucleoside transporter (ENT)1 and ENT2, which acts on presynaptic A_1A _receptor to suppress neurotransmitter release, whilst increased neuronal activity activates the astrocyte A_2B_ receptor to increase lactate provision for neuronal mitochondrial metabolism [75,76]. Astrocytes regulate CNS adenosine via the expression of a cytoplasmic ‘short’ and nuclear ‘long’ form of adenosine kinase, which converts adenosine to AMP [77]. Overall, ketamine effects may be primarily on astrocytes, astrocyte mitochondria and astrocyte regulation of adenosine, with significant consequences for astrocyte and other CNS cells mitochondrial function as well as neuronal activity and survival.

Astrocytes in the PFC, especially the mPFC, show significant changes in MDD and preclinical models, which is characterized by changes in morphology, reduced density, and alterations in signaling, such as suppressed ATP release and dysregulated Ca^2+ ^signaling. Such alterations in astrocytes change their regulation of neuronal activity, including glutamatergic and serotonergic regulation as well as synaptic plasticity and metabolic support for neurons. This is evident in preclinical corticosteroid driven stress models [78] as well as clinically [79,80]. Both optogenetic and chemogenetic activation of mPFC astrocytes, which enhance astrocyte mitochondria Ca^2+^ influx, negate the impacts of developmental stress-driven depression and increase the social rank of subordinate rodents and change the neuronal excitatory/inhibitory ratio in the mPFC, including by increasing astrocyte EAAT levels [81]. The dysregulated glutamatergic activity in the MDD PFC is therefore powerfully determined by alterations in astrocyte function, suggesting that ketamine’s efficacy as an antidepressant is more likely to be mediated via astrocyte function and downstream neuronal regulation than by its inhibition of the post-synaptic NMDAR [20].

Notably, in preclinical models of stress driven depression, there is a selective increase in BBB permeability in the mPFC [82], which may be contributed to by dysregulated astrocyte function and may preferentially enhance ketamine entry into the mPFC. Clinical studies also indicate increased BBB permeability in the hippocampus, white matter and thalamus in MDD patients that positively correlate with depression severity [83]. Ketamine has increased entry into the brain at sites with increased BBB permeability, as indicated by ketamine dose at anesthetic levels, with ketamine entry into the CNS decreasing BBB permeability [84]. Consequently, ketamine levels and effects may be heightened at sites of increased BBB permeability, with impacts on astrocyte function that contribute to re-establishing neuronal function and the BBB [85]. This re-establishment of the BBB and astrocyte regulated neuronal activity alters the inter-area communication across the brain.

**Fig. 2.**
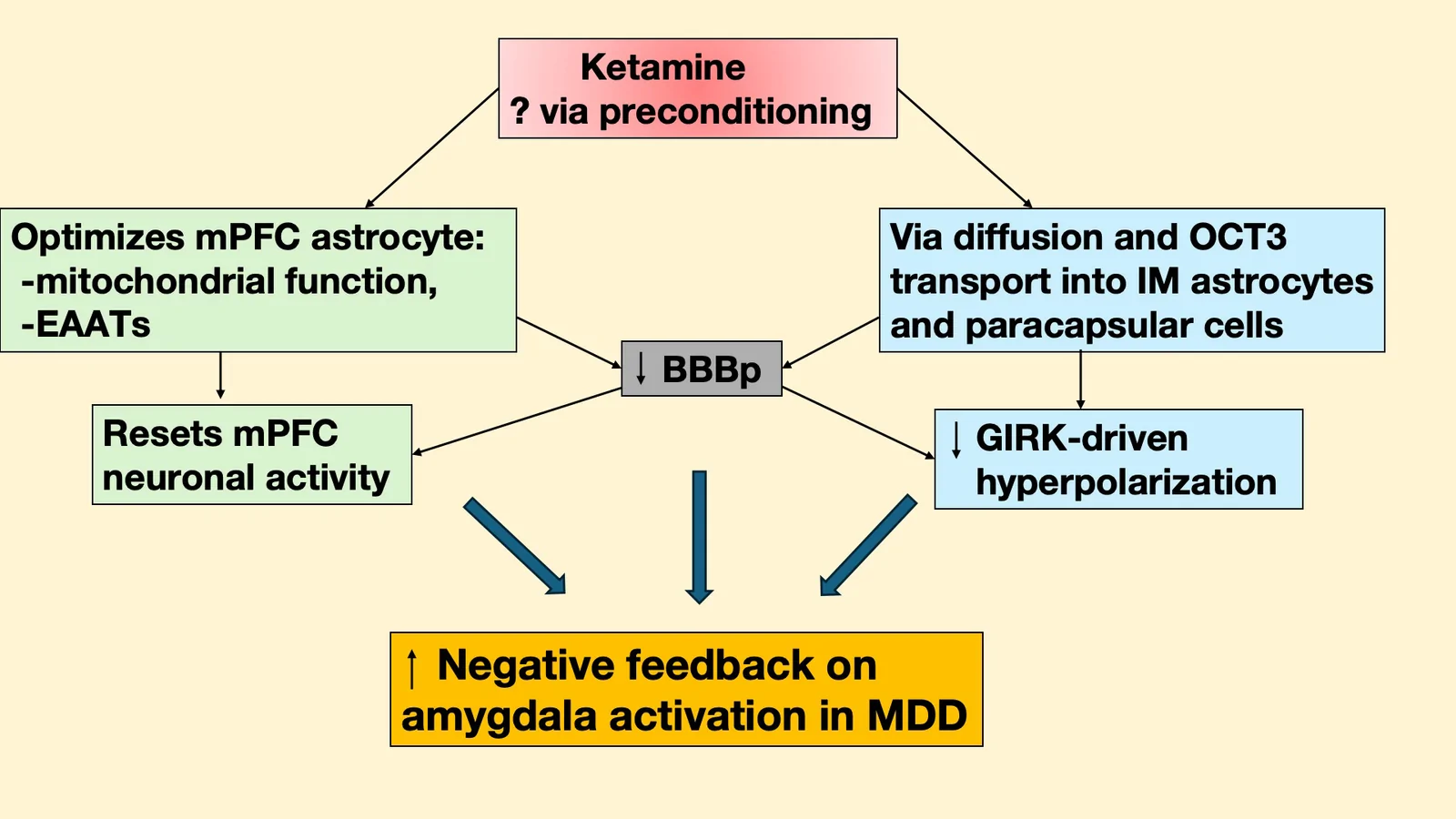
**Ketamine acts at multiple sites to modulate mood**. Shows ketamine to act at mPFC astrocytes to precondition mitochondria to optimize mitochondrial function and EAATs (green shade), which resets mPFC neuronal activity to negatively feedback on the heightened amygdala activation that is typical in MDD. For this to occur ketamine may simultaneously act, via diffusion and/or OCT3 driven uptake, on the astrocytes and paracapsular cells of the intercalated masses to decrease GIRK-driven hyperpolarization, thereby allowing PFC neuronal activity to negatively feedback on heightened amygdala activation. These processes may be potentiated by ketamine increasing BBB integrity either directly and/or via astrocyte mitochondria regulation. Abbreviations: BBBp, blood-brain barrier permeability; EAAT, excitatory amino acid transporter; GIRK, G-protein-gated inwardly rectifying K^+^ channel; IM, intercalated masses; mPFC, medial prefrontal cortex; OCT3, organic cation transporter 3. ?: indicates that requires experimental validation.

The re-establishment of the BBB and neuronal activity may be particularly pertinent in the mPFC where a suppressed capacity by the PFC to negatively feedback on amygdala function has long been proposed to increase the affective modulation of cognition underpinning MDD [86] and other psychiatric conditions [37]. Increased amygdala BBB permeability is also evident in preclinical models of stress-induced depression [87]. Heightened and dysregulated amygdala activity in MDD may be partly mediated by increased dopamine at the amygdala dopamine (D)1r, including in the paracapsular cells of the intercalated masses where D1r activation leads to paracapsular cell hyperpolarization via increased G-protein-gated inwardly rectifying K^+^ channel (GIRK) activation that prevents negative feedback from the PFC to the amygdala [37]. Astrocytes typically express the dopamine D1r and, via ionic regulation, modulate hyperpolarization [88]. Interestingly, OCT3 is highly expressed in the intercalated masses [89], suggesting that there may be increased ketamine active transport into these cells and associated astrocytes to alter their mitochondrial function and regulation of PFC feedback to the amygdala, see Fig. 2. Overall, ketamine may have heightened effects in astrocyte mitochondria at sites of increased BBB permeability, thereby contributing to BBB integrity [85] and the re-establishment of interarea neuronal communication, including PFC-amygdala interactions that are pertinent to mood, emotion and stress regulation, shown in Fig. 2.

Importantly, the NMDAR present on the inner mitochondrial membrane can be regulated by NMDAR agonists and other non-competitive NMDAR antagonists, such as (+)-5-methyl-10,11-dihydro-5H-dibenzo[a, d]cyclohepten-5,10-imine (MK-801). Mitochondrial NMDAR activation increases Ca^2+^ influx, indicating that ketamine may act at this receptor to suppress Ca^2+^ influx, thereby affording protection under stress-linked Ca^2+^ influx upregulation [90]. Paralleling the proposed effects of ketamine post-synaptically, where ketamine’s inhibition of the NMDAR alters ionic regulation by increasing AMPAr and associated lower level, less toxic Ca^2+^ influx that increases mechanistic target of rapamycin (mTOR) to modulate mitochondrial function [27,28], ketamine effects on mitochondrial NMDAR also changes mitochondrial ionic regulation. In principle, ketamine can diffuse or be OCT3 transported over the astrocyte mitochondrial bilipid membrane [91] to modulate the inner mitochondrial membrane NMDAR. The initial transport over the BBB to astrocytic end-feet indicate that ketamine uptake in astrocytes (and subsequently neurons) is more likely to lead to mitochondrial uptake rather than interacting with the more distal synaptic and peri-synaptic astrocyte and neuronal NMDAR. Direct effects of ketamine and its metabolites on mitochondria have been shown previously, although this has primarily been investigated at high ketamine concentrations that lead to mitochondrial toxicity by decreasing the mitochondrial electron transport system and ATP production, as well as increasing caspases, reactive oxygen species (ROS) and mitochondrial fission [92]. These authors also showed ketamine to impair Complex I, leading to decreased nicotinamide adenine dinucleotide plus hydrogen (NADH) consumption during OXPHOS and associated decrease in conversion to NAD^+^ [92], thereby negatively impacting on NAD^+^ dependent sirtuins. In contrast, low dose ketamine increases astrocyte glucose uptake, mitochondrial ATP and lactate provision for neurons [93]. Data by Yue and colleagues [20] shows relatively low doses of ketamine to act on isolated neuronal mitochondria to regulate adenosine release, which is proposed here to be mediated by ketamine modulation of astrocyte mitochondria.

There is a growing interest in how classically conceptualized neurotransmitters can directly modulate mitochondrial function, as indicated by the presence of diverse receptors on the mitochondrial membrane, including melatonin (MT)1r and MT2r, GR-α, endocannabinoid receptor (CB)1r, and CB2r, the BDNF/NAS receptor, TrkB, as well as classical transcription factors, such as mitochondria translocating NF-κB and STAT3 [94]. All of these receptors and transcription factors significantly modulate mitochondrial function [94]. The effects of low dose ketamine on the mitochondrial NMDAR and mitochondrial function requires fuller investigation, including the relevance of ketamine as a positive allosteric modulator of mitochondrial TrkB (see Fig. 1). Data to date on the toxic effects of ketamine at higher doses in mitochondria would suggest that the typically lower ketamine doses and relatively short half-life and exposure time in MDD treatment may act to precondition mitochondria. As noted, preconditioning is a common process across diverse forms of life, whereby exposure to a mild, non-lethal stress/stimulus, leads to protective changes/mechanisms that make an animal/plant/fungi more resistant to any subsequent more severe stress [95].

**Fig. 3.**
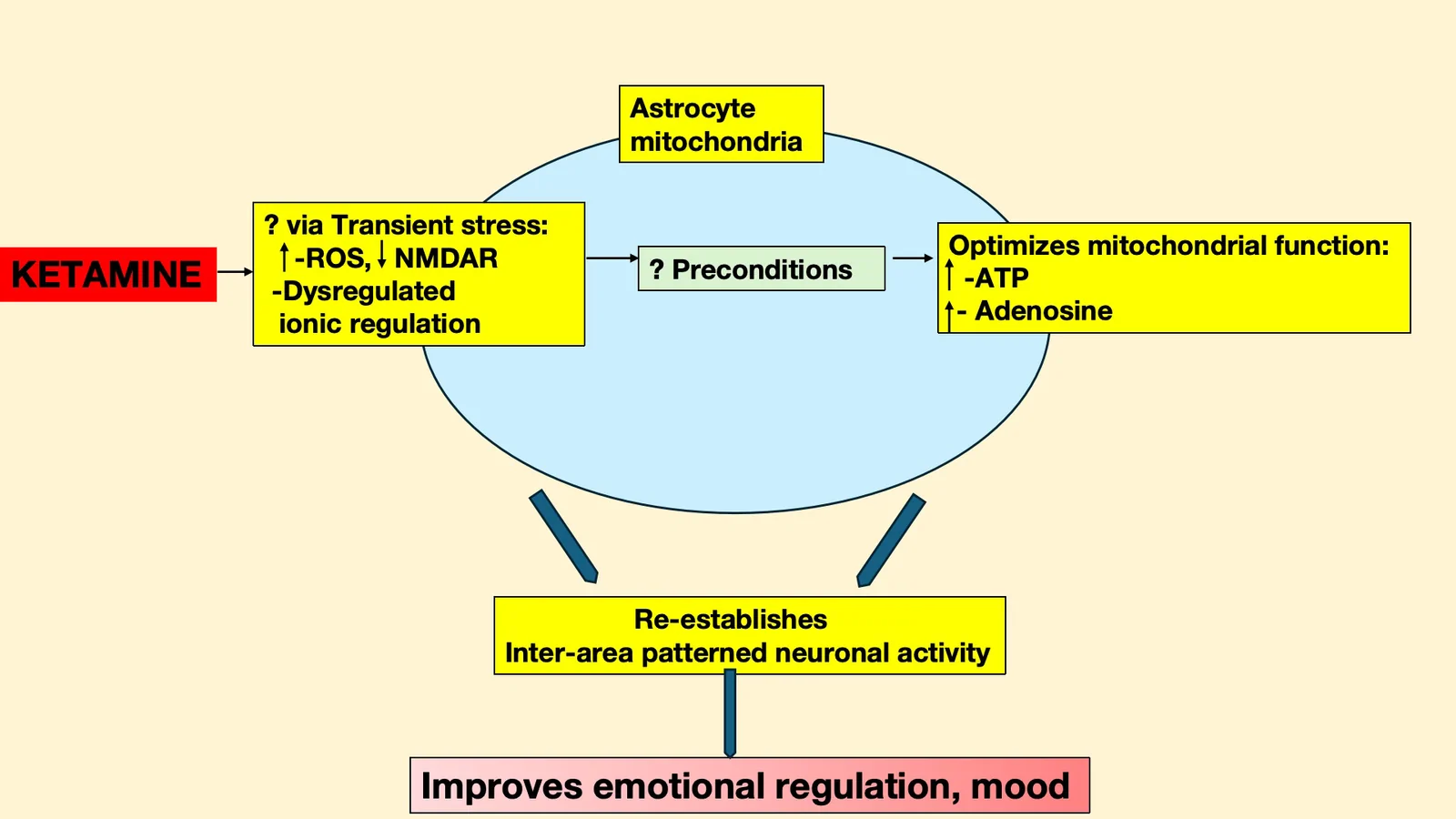
**Ketamine has transient stress effects**. Shows ketamine to have initial, transient stress effects, which preconditions astrocyte mitochondria to more optimal function, including increasing ATP and adenosine as well as wider astrocytic functions that ultimately re-establishes inter-area patterned neuronal activity that improves emotional regulation and mood. Abbreviations: ATP, adenosine triphosphate; ROS, reactive oxygen species. ?: indicates that requires experimental validation.

This raises a number of questions. Does a single ketamine dose have preconditioning effects on mitochondrial function? Is such putative preconditioning mediated via ketamine having inhibitory effects on the mitochondrial inner membrane NMDAR capacity to restrict Ca^2+^ entry in mitochondria when coupled to TrkB activation and/or positive allosteric TrkB modulation? Does this Ca^2+^ dysregulation underpin adenosine upregulation, leading to a cAMP/PKA initiation of the mitochondrial melatonergic pathway? Does mitochondria NMDAR inhibition lead to compensatory changes in wider mitochondrial ionic regulation e.g., preconditioning effects on the levels of Mitochondrial calcium uniporter (MCU), leucine zipper and EF-hand containing transmembrane protein 1 (LETM1) domain-containing protein 1 (LETMD1)/LETM1 (which share a structural sequence domain), or pSTAT3^Ser727^ at MAMs, where pSTAT3^Ser727 ^down-regulates Ca^2+^ influx from the endoplasmic reticulum [96]. Wider ionic regulation is given some support by data showing ketamine to induce a ROS-dependent rise in MCU activity and Ca^2+^ influx [97]. Does the proposed putative preconditioning effects of ketamine on astrocyte mitochondria also arise from increased ROS that drives ROS-dependent miRNAs that shape patterned gene expression in the nucleus and/or mitochondria? Would this include alterations in glutamatergic receptors, such as NMDAR subunits and NMDAR expression on the mitochondrial inner membrane and/or the increased plasma membrane AMPAr levels that are induced by ketamine? Overall, given ketamine’s amphiphilic nature and astrocyte proximity to the BBB, ketamine’s first effects in the CNS are more likely at astrocyte mitochondria and then astrocyte NMDAR, before entering and modulating the neuronal plasma membrane and mitochondrial NMDAR. Whether this follows a preconditioning paradigm will be important to determine. See Fig. 3.

## 4. Astrocyte Humanin and Glia Melatonin

A number of processes may show alteration in astrocytes under such putative mitochondria preconditioning type effects. There is a growing interest in the role of mitochondria-derived peptides, such as humanin, in the regulation of diverse CNS conditions, including neurodegenerative conditions [98] and neuropsychiatric conditions, such as MDD, where humanin is significantly decreased [99]. Antidepressant efficacy and the resolution of MDD pathophysiology are strongly determined by increasing humanin levels [100]. Humanin is primarily produced and released by astrocytes in the CNS [101]. Humanin has intracellular (blocks Bax and Bid mitochondrial translocation/apoptosis) and intercellular (shifting of microglia from an M1-like to M2-like phenotype) effects [102,103,104,105]. As shifting to an M2-like microglia phenotype requires changing NF-κB dimers from p65/p50 to a c-Rel/p50 and subsequent upregulation, release and autocrine effects of melatonin [106], astrocyte humanin’s intercellular effects may include paracrine effects that upregulate the mitochondrial melatonergic pathway and melatonin release in microglia, neurons and/or oligodendrocytes. There is no data to indicate that humanin increases the cAMP/PKA/aralkylamine N-acetyltransferase (AANAT)/melatonergic pathway. However, another mitochondria-derived peptide, mitochondrial ORF of the 12sRNA Type C (MOTS-c) can increase the cAMP/PKA pathway [107] and requires investigation whether it is regulated by ketamine in astrocytes and whether it is released to increase the melatonergic pathway in microglia and neurons.

The capacity of ketamine (and ketamine induced adenosine) to increase cAMP/PKA and local melatonin [25] may therefore involve an initial upregulation and release of mitochondria-derived humanin and/or MOTS-c. This is supported by data showing humanin to increase STAT3 [101], which interacts with variations in NF-κB dimer composition to up- or down-regulate the melatonergic pathway [108]. Humanin extracellular effects are mediated via a variety of different receptors, including G protein-coupled receptors, formyl peptide-receptor-like (FPRL)-1 and 2; a trimeric receptor complex comprised of ciliary neurotrophic factor receptor α (CNTFr-α), gp130, and the interleukin (IL)-27 receptor subunit, IL-27Rα; as well as the gp130 receptor, the presence of which may vary across cell types [109]. How these different receptors link to NF-κB dimer composition and melatonergic pathway regulation in different cells will be important to determine.

Humanin is also protective of oligodendrocytes [110] and therefore protects against the white matter damage that can occur in MDD, which has been proposed to drive MDD pathogenesis [111]. Although microglia are typically conceptualized as the major drivers of neuro-inflammation and therefore a significant treatment target, astrocytes, via humanin and humanin-induced melatonin upregulation, may be crucial determinants of neuro-inflammation resolution via microglia phenotype regulation in both grey and white matter.

**Fig. 4.**
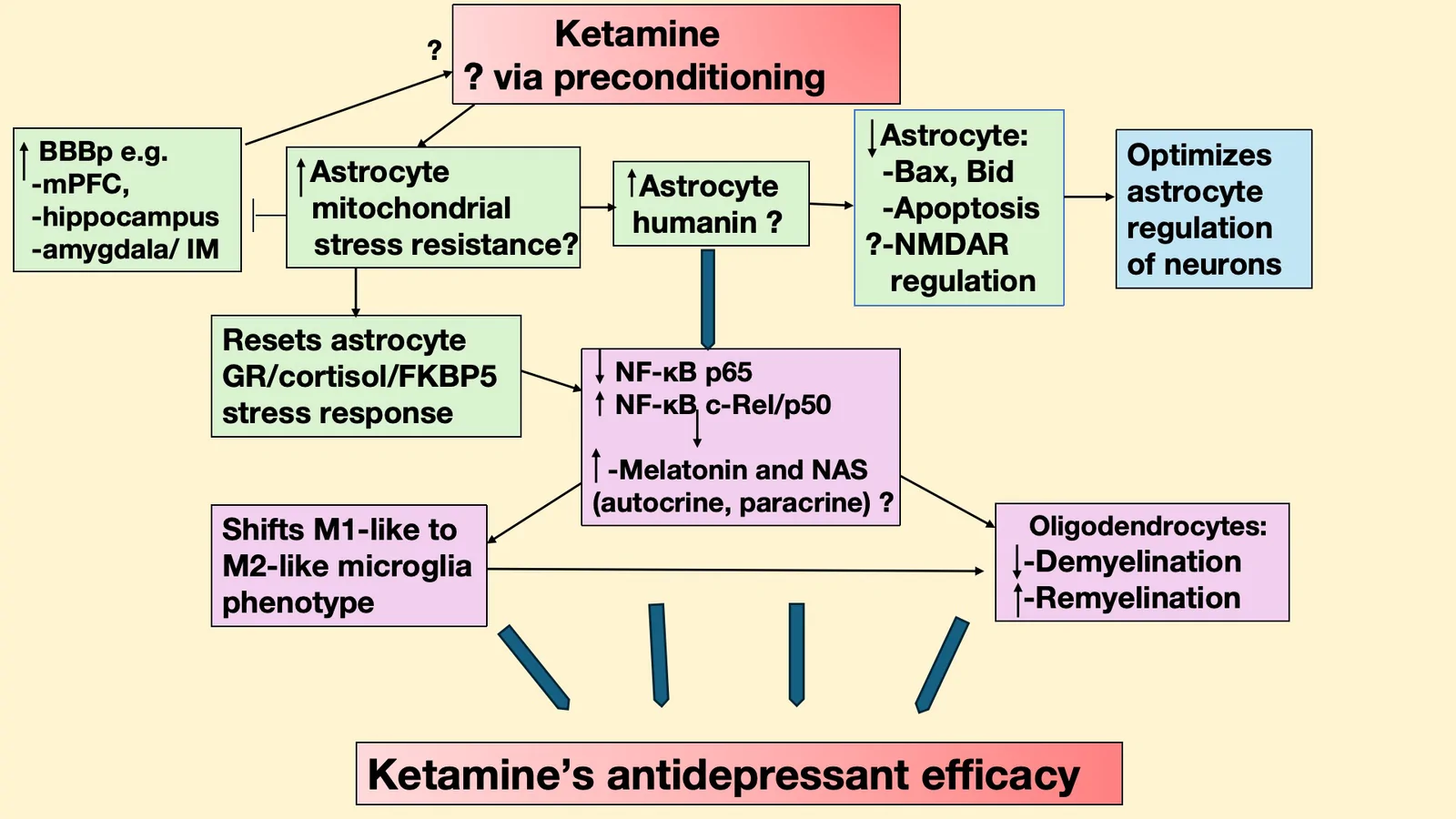
**Ketamine preconditions astrocytes to increase stress resilience**. Shows ketamine preconditioning to increase astrocyte ‘stress resilience’, leading to increased astrocyte humanin, which has intracellular effects by inhibiting Bax, Bid and apoptotic processes as well as possible astrocyte and neuronal NMDAR regulation, with these effects potentiated at sites of increased BBB permeability (green shade), where ketamine has enhanced entry and effects. The increased astrocyte stress resistance resets the influence of developmental stressors/trauma. These changes also optimize astrocyte regulation of neuronal function (blue shade). Astrocyte humanin also has extracellular effects including on microglia and oligodendrocytes, where humanin decreases NF-κB p65 and increases NF-κB c-Rel/p50, which can upregulate the melatonergic pathway (as shown in other cell types) and therefore increase the autocrine and paracrine effects of melatonin and possibly NAS. Autocrine melatonin shifts microglia to a quiescent M2-like phenotype, with humanin and the M2-like microglia increasing remyelination and decreasing demyelination (purple shade). These effects of ketamine-driven humanin on mitochondrial function and associated cell phenotypes in astrocytes, microglia and oligodendrocytes are proposed to underpin its antidepressant efficacy. Abbreviations: Bax, Bcl-2-associated X protein; Bid, BH3-interacting domain death agonist; FKBP5, Fujisawa code Kuroda/Kino 506 (FK506)-binding protein 5; NF-κB, nuclear factor kappa-light-chain-enhancer of activated B cells. ?: indicates that requires experimental validation.

Whether the putative preconditioning effects of ketamine increases humanin and thereby local melatonin will be important to determine. Preconditioning (transient mild stress increasing resilience) is a pattern that enhances humanin production under other ‘stressor conditions’, such as the dampening of inflammation or oxidative stress [112]. Whether such changes in astrocyte mitochondrial function, especially at sites of increased BBB permeability such as the mPFC, underpins the maintained improved mood following ketamine administration also requires investigation. Humanin, like ketamine and MK-801, also attenuates NMDAR overactivation/excessive Ca^2+^ influx and ROS production [2,5,109], suggesting that increased humanin production in ketamine preconditioned astrocyte mitochondria may underpin the continued attenuation of NMDAR over-activation in ketamine’s absence [113]. Fig. 4 highlights ketamine effects in astrocytes, which is parsimonious with recent studies highlighting the role of alterations in astrocyte function in MDD [21,114,115].

Ketamine may also act directly on microglia (not shown in Fig. 4 for clarity). Activated microglia in MDD enhance pro-inflammatory cytokines levels and synaptic pruning, leading some to regard MDD as a ‘microgliopathy’ [116,117]. Preclinical data shows stress-induced depression to be intimately associated with a pro-inflammatory M1-like microglia phenotype [118,119]. Microglia activation also contributes to demyelination and suppressed remyelination that is also evident in depression [111,120], whilst the conversion to an M2-like microglia phenotype by ketamine directly and/or indirectly via astrocyte derived humanin and melatonin increases remyelination [121]. These authors propose that increased remyelination underpins ketamine’s sustained antidepressant effects. Ketamine rapidly upregulates microglia pSTAT3^Tyr705 ^[122] and decreases pro-inflammatory NF-κB p65 partly by increasing sirtuin-2 driven deacetylation [123], indicating alterations in wider cellular factors/processes that are compatible with upregulating the microglia melatonergic pathway [108]. Ketamine may also act via systemic processes including the gut microbiome to modulate microglia activation [124], including from increased gut microbiome derived, butyrate [3], which shifts microglia from an M1-like to M2-like phenotype [125]. Overall, ketamine can significantly modulate microglia function directly and indirectly (via the gut or astrocytes) to dampen MDD pathophysiology.

## 5. Reframing Classical Ketamine Modulation of Mitochondria

Ketamine’s mitochondrial modulation is classically modeled as arising from post-synaptic NMDAR inhibition/negative allosteric inhibition [126,127] that either (a) decreases Ca^2^/ mitogen activated protein kinase (MAPK) pathway that alters ATP synthase catalytic subunits to decrease mitochondrial ATP synthesis [92]; or (b) alters the Ca^2+^/calmodulin-dependent protein kinase II (CaMKII)/extracellular signal-regulated kinase (ERK) pathway as driven by MK-801 [128]. At higher concentrations, ketamine can suppress mitochondrial complex I (NADH dehydrogenase) and complex V (ATP synthase), thereby enhancing the NADH/NAD+ ratio and inhibiting metabolic efficiency [92]. Rather, it is proposed here that low dose ketamine’s regulation of mitochondrial function may be directly at the inner mitochondrial membrane NMDAR and outer mitochondrial membrane TrkB, with preconditioning ionic regulatory effects that upregulate the mitochondrial resilience of astrocytes and neurons. This negates the effects of developmental stress/trauma via cortisol/GR-α on astrocytes and parallels the effects of other novel antidepressants, such as cannabidiol, which are also mediated via modulating mitochondrial function [129].

## 6. Discussion and Implications

The above has a number of wider implications, including in how mood/depression and long-term potentiation (LTP)/long-term depression (LTD) are conceptualized. It also links wider bodies of physiological data associated with depression, including regulation of the opioidergic system, and oxytocin interactions with stress as well as vagal nerve alterations and interactions with systemic inflammation.

### 6.1 Reconceptualizing Mood/Depression

Ketamine’s direct regulation of mitochondria not only provides a model of its fast-acting antidepressant efficacy but also highlights the importance of astrocytes and astrocyte mitochondria in determining subjective mood/emotional state. The ‘shotgun’ effects of ketamine at sites of heightened BBB permeability suggest that it has heightened effects at sites where astrocytes and microglia are struggling to resolve local dysregulation, thereby increasing BBB permeability to allow immune cells entry if required, as evident in neurodegenerative conditions [98]. It is proposed that inter-area patterned neuronal activity alterations across the CNS emerge from developmental/childhood stress/trauma that act via enhanced cortisol and GR-α activation to modulate astrocyte mitochondria and therefore astrocyte function [130,131], leaving an epigenetic trace that makes some CNS regions susceptible to subsequent stressors [130,132]. By optimizing astrocyte function the fast-acting antidepressants re-establish the BBB, debris collection by the glymphatic system, EAATs and lactate provision for neurons to re-establish neuronal function. This clearly describes a novel conceptualization of depression and stress-regulated brain processes more generally, indicating that treatment of depression focuses on optimizing astrocyte mitochondrial function, possibly specifically at sites of increased BBB permeability. This has distinct testable treatment implications.

### 6.2 Implications for LTP/LTD

Ketamine also regulates fundamental cognitive processes, namely LTP and LTD [133,134], partly mediated via ketamine’s bioactive metabolite, (2R,6R)-HNK [135]. Ketamine’s regulation of LTP and LTD is also classically modeled via NMDAR inhibition, primarily on hippocampal GABAergic interneurons thereby attenuating their inhibition of glutamatergic neuronal plasticity [130,131]. However, ketamine effects on astrocyte mitochondria to increase humanin and the melatonergic pathway may be more parsimonious with wider bodies of data on LTP/LTD. A growing body of preclinical data shows hippocampal LTP and LTD to be highly dependent upon the capacity to release melatonin locally in the hippocampus [136]. Hippocampal AANAT initiates the melatonergic pathway to optimizes the induction of LTP, cognition and memory [137].

As noted, the immediate precursor of melatonin, NAS, activates the BDNF receptor, TrkB [56] and induces hippocampal BDNF [57], as can humanin [138], with TrkB-FL activation a core aspect of neurogenesis [139,140] and LTP [141]. Ketamine’s capacity to upregulate the melatonergic pathway may therefore be an unrecognized route for its regulation of hippocampal LTP, LTD and neurogenesis. The regulation of the NAS/melatonin ratio, e.g. by ketamine increasing mitochondrial ATP to activate the purinergic (P)2Y1 receptor, which increases the NAS/melatonin ratio [142] and may be an unrecognized aspect of LTP, LTD and neurogenesis [143]. This is given indirect support by data showing adjunctive melatonin to potentiate ketamine effects on neurogenesis and cognition [144]. As astrocytes and astrocyte mitochondrial function are major regulators of synaptic plasticity [145] and neurogenesis [146], ketamine effects on astrocyte mitochondria are highly suggestive of a role for induced melatonin and humanin in LTP/LTD and requires investigation.

### 6.3 Integrating the Opioidergic System, TrkB, Oxytocin Interactions With Stress and Vagal Nerve Interactions With Systemic Inflammation

Ketamine is also an allosteric modulator of opioidergic receptors [147], as well as inducing pro-opiomelanocortin (POMC) to increase β-endorphin [148], thereby modulating LTP and LTD [149]. As the opioidergic system is increasingly recognized as a powerful regulator of mood [150], ketamine effects on the opioidergic system contribute to its antidepressant efficacy. Whether this is mediated via initial effects on astrocyte mitochondria, given the role of astrocytes in the regulation of the opioidergic system [151], will be important to determine. Notably, ketamine’s antidepressant efficacy in a preclinical model of adolescent stress that increases subsequent adult stress-driven depression, is potentiated by the adjunctive use of a kappa-opioid receptor antagonist [152], indicating the utility of induced and/or adjunctive melatonin, which can decrease kappa-opioid receptor expression [153].

As noted, ketamine also shows positive allosteric modulation of the BDNF receptor, TrkB, which contributes to LTP as well as to ketamine’s longer-term antidepressant efficacy [154]. As TrkB, predominantly the truncated TrkB-T1, is expressed on astrocyte mitochondria where it regulates mitochondrial function [155], ketamine’s antidepressant effects and modulation of LTP/neuronal plasticity may be modulated via astrocyte mitochondrial TrkB-T1 [156]. Consequently, astrocyte mitochondrial membranes provide two sites on which ketamine can modulate mitochondrial and wider cellular/intercellular processes, namely the NMDAR on the inner mitochondrial membrane and TrkB on the outer mitochondrial membrane.

Decreased oxytocin is often evident in depression, especially in severe depression [157]. Oxytocin projections from the hypothalamic PVN to the central amygdala (CeA) act on CeA astrocytes to prevent stress-induced cortisol activation of the GR-α from inducing local CRH and therefore the CRH induction of dynorphin and kappa-opioid receptor activation, which in the basolateral amygdala (BLA) increases dysphoria [150]. Similar interactions of CRH and the opioidergic system occur in the hippocampus (cognition) and ventral tegmental area (VTA)/nucleus accumbent (N.Acc) (motivation), where ketamine and melatonin’s capacity to upregulate β-endorphin and mu-opioid receptor activation shifts the balance of the mu-/kappa-opioid receptor ratio to improve mood/affect, cognition and motivation.

**Fig. 5.**
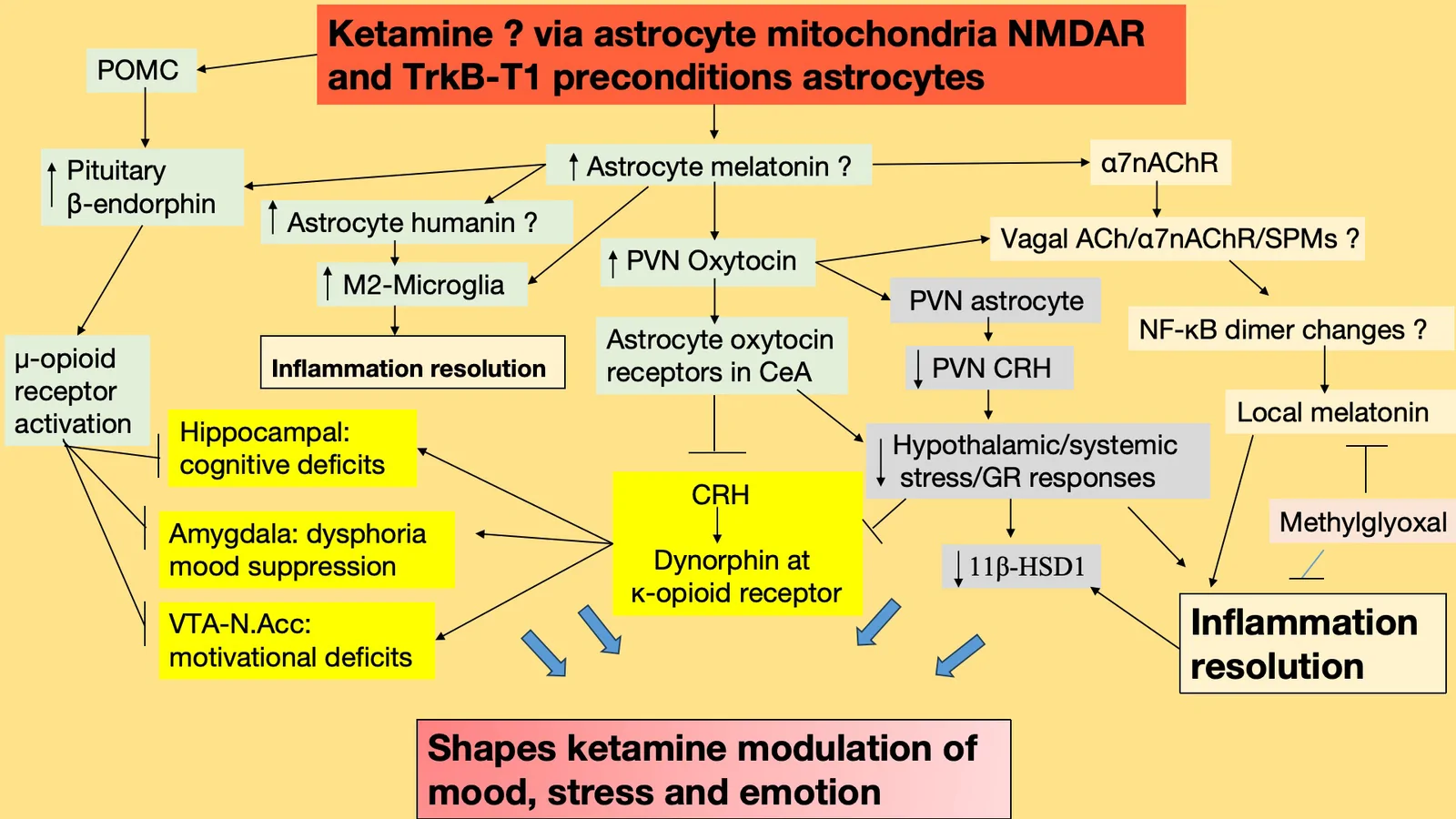
**Ketamine, via astrocyte mitochondrial NMDAR and TrkB-T1 preconditions astrocytes to regulate mood, stress and emotion**. This is partly mediated via the oxytocin and opioidergic systems and the capacity of the vagal nerve to achieve inflammation resolution. Ketamine is proposed to precondition mitochondria, thereby upregulating the mitochondrial melatonergic pathway systemically but with special relevance in astrocytes. This may be mediated via STAT3 and NF-κB interactions (not shown for clarity) as well as ketamine’s induction of cAMP/PKA [167], which is classically associated with initiating the melatonergic pathway [168]. Melatonin may therefore be an aspect of the ‘bounce back’ of preconditioning that subsequently upregulates humanin, oxytocin and β-endorphin (green shade). PVN oxytocin neurons project to CeA astrocyte receptors to dampen stress/GR-α induction of CeA CRH, thereby attenuating the basolateral amygdala (BLA)/dynorphin/kappa-opioid receptor/dysphoria pathway [169] as well as dynorphin effects on hippocampal cognition and VTA/N.Acc motivation (yellow shade). Ketamine increases POMC [148] and melatonin, which both increase β-endorphin. β-endorphin acts on the mu-opioid receptor, thereby suppressing deficits in the hippocampus (cognition), amygdala (affect) and VTA/N.Acc (motivational). Ketamine is proposed, possibly via melatonin, to upregulate mitochondrial humanin, with released astrocyte melatonin and humanin shifting the pro-inflammatory M1-like microglia to a more quiescent M2-like phenotype, thereby contributing to local microenvironment inflammation resolution. Acute ketamine, via melatonin, PVN oxytocin and the opioidergic system thereby alters inter-area patterned neuronal activity, including via the circadian and stress induction of the HPA axis and GR-α activation. This alters the nature of the stress response and counter-stress resolution processes (grey shade). Increased GR-α activation, especially in the presence of the raised pro-inflammatory cytokines evident in depression, upregulates local cellular cortisol production by 11β-HSD1 that contributes to wider pathophysiological alterations. Adjunctive melatonin and/or oxytocin may therefore attenuate ketamine side-effects especially when acute ketamine is unable to upregulate local melatonin or PVN oxytocin. Inhibitors of the tryptophan-melatonin pathway, such as methylglyoxal (orange shade), therefore modulate ketamine effects as well as the capacity of vagal nerve activation to achieve inflammation resolution. Abbreviations: 11β-HSD1, 11β-hydroxysteroid dehydrogenase 1; α7nAChR, alpha 7 nicotinic acetylcholine receptor; ACh, acetylcholine; BLA, basolateral amygdala; cAMP, cyclic adenosine monophosphate; CEA, central amygdala; CRH, corticotropin-releasing hormone; HPA, hypothalamic-pituitary-adrenal; N.Acc, nucleus accumbens; PKA, protein kinase A; POMC, pro-opiomelanocortin; PVN, hypothalamic paraventricular nucleus; SPMs, specialized pro-resolving mediators; VTA, ventral tegmental area. ?: indicates that requires experimental validation.

Acute, but not chronic, ketamine increases oxytocin [158], whilst ketamine induction of melatonin [25] increases oxytocin [159]. Oxytocin [160], and in some circumstances melatonin [161], enhance vagal nerve activation, the effects of which are heightened by melatonin’s upregulation of the alpha 7 nicotinic acetylcholine receptor (α7nAChR) [162], upon which vagal ACh acts to dampen inflammation. Notably, the efficacy of vagal stimulation to dampen inflammation, preclinically and clinically, is dependent upon the capacity to upregulate local melatonin [163,164], suggesting that ketamine may not only enhance vagal stimulation but, by increasing local melatonin [25], also determine vagal efficacy in dampening systemic inflammation. The concurrent effects of ketamine at different systemic sites may therefore interact to modulate its capacity to dampen systemic inflammation and how these systemic effects modulate BBB permeability and CNS function [165]. By upregulating oxytocin, acute ketamine, and ketamine induced local melatonin, decrease PVN CRH induction, thereby suppressing both pro-inflammatory cytokine and excessive GR-α activation that induce 11 beta-hydroxysteroid dehydrogenase (11β-HSD1). 11β-HSD1 is a genetic risk factor for suicide attempts and poor antidepressant response [166], suggesting that the downstream inhibition of 11β-HSD1 by ketamine/melatonin/oxytocin has clinical relevance. See Fig. 5 (Ref. [148,167,168,169]).

It will be important for future research clarify any differential physiological processes underpinning the distinct effects of (S)-ketamine [3,123], (R)-ketamine [124] and hydroxyketamine [170]. Preclinical data indicates that R-ketamine produces more potent and longer-lasting antidepressant effects than S-ketamine [171], leading to R-ketamine being proposed to have treatment efficacy across diverse medical presentations, including neurodegenerative conditions [172]. Whether this is mediated by distinct (R)-ketamine effects on astrocytes and astrocyte mitochondria, which are increasingly recognized as important pathophysiological aspects and treatment targets of neurodegenerative conditions [33], will be important to clarify. As (S)-ketamine has stronger binding affinity at the NMDAR [173], this may suggest that the increased antidepressant efficacy of (R)-ketamine may arise from relatively enhanced effects at other sites, such as the mitochondrial TrkB or other modulators of mitochondrial function including adenosine [20] at the mitochondrial adenosine A3 receptor [174]. This will be important to clarify in future research.

The importance of mitochondrial function to ketamine effects are highlighted by its antidepressant efficacy being linked to increased mitochondrial oxidative phosphorylation in the rostral anterior cingulate, as shown in clinical adolescent depression [74,175]. This is supported by longer standing preclinical data showing ketamine to increase mitochondrial oxidative phosphorylation [176]. Clearly, this requires investigation in astrocytes as well as in other CNS cells, including as to any differential effects of (S)-ketamine vs (R)-ketamine.

It will also be important to clarify the relevance of direct effects of ketamine and its enantiomers on microglia BDNF production and release [177] as well as the production and release of the immediate melatonin precursor NAS, which is a BDNF mimic [56].

## 7. Conclusions

Ketamine’s direct regulation of mitochondria provides not only an understanding of its fast-acting antidepressant efficacy but also says something fundamental about the core pathophysiological changes underpinning mood regulation and the subjective state of depression. It is proposed that ketamine effects in astrocyte mitochondria across diverse brain regions, including the mPFC, amygdala, hippocampus and white matter allows ketamine to reset many of the astrocyte changes occurring in depression, including neurotransmitter uptake and redistribution, synaptic regulation and lactate provision for neighboring neurons. Ketamine has more ready access to astrocytes at sites of increased BBB permeability, which includes classical sites associated with depression. Ketamine effects on astrocyte mitochondria seem mediated via the NMDAR on the astrocyte (and neuronal) inner mitochondrial membrane and the TrkB-T1 on the astrocyte mitochondrial membrane, which alter astrocyte ionic regulation, with the transient stressor effects of ketamine acting to precondition mitochondria. The optimization of astrocyte mitochondrial function following stress increases humanin and melatonin that have intracellular and extracellular effects, which dampen microglia activity, increase remyelination, and re-establish neurotransmitter uptake, recycling and release, thereby allowing more typical cellular communication from these brain sites, such as the mPFC and amygdala, which, in turn, change patterned inter-area communication across the brain.
